# Total synthesis of ochnaflavone

**DOI:** 10.3762/bjoc.9.152

**Published:** 2013-07-08

**Authors:** Monica M Ndoile, Fanie R van Heerden

**Affiliations:** 1 School of Chemistry and Physics, University of KwaZulu-Natal, Private Bag X01, Scottsville 3209, Pietermaritzburg, South Africa

**Keywords:** biflavone, diaryl ether, natural products, nucleophilic aromatic substitution, ochnaflavone, tetrahydroochnaflavone

## Abstract

The first total syntheses of ochnaflavone, an asymmetric biflavone consisting of apigenin and luteolin moieties, and the permethyl ether of 2,3,2'',3''-tetrahydroochnaflavone have been achieved. The key steps in the synthesis of ochnaflavone were the formation of a diaryl ether and ring cyclization of an ether-linked dimeric chalcone to assemble the two flavone nuclei. Optimal experimental conditions for the oxidative cyclization to form ochnaflavone were established.

## Introduction

Biflavonoids are a class of compounds that are receiving increasing attention because of their biological activity. Biflavonoids with anti-inflammatory [1–3], antileishmanial [4–5], antiplasmodial [6–8], antiviral [9] and β-secretase inhibitory [10] activity, amongst others, have been reported. The activity of the biflavonoids is in general much higher than that of the individual monomers [11]. The flavonoid units in these compounds are joined in a symmetric or asymmetric manner and can either be identical or nonidentical. In addition, the two flavonoid units can be linked through a C–C bond or C–O–C bond. Many biflavonoids consist of an interflavonoid linkage between ring B of one moiety and the ring A of the other moiety (AB type), between two A rings (AA type) or between the two C rings (3,3''-CC type), but the most rare biflavonoids are the ones with the interflavonoid linkage between the two B rings.

**Figure 1:**
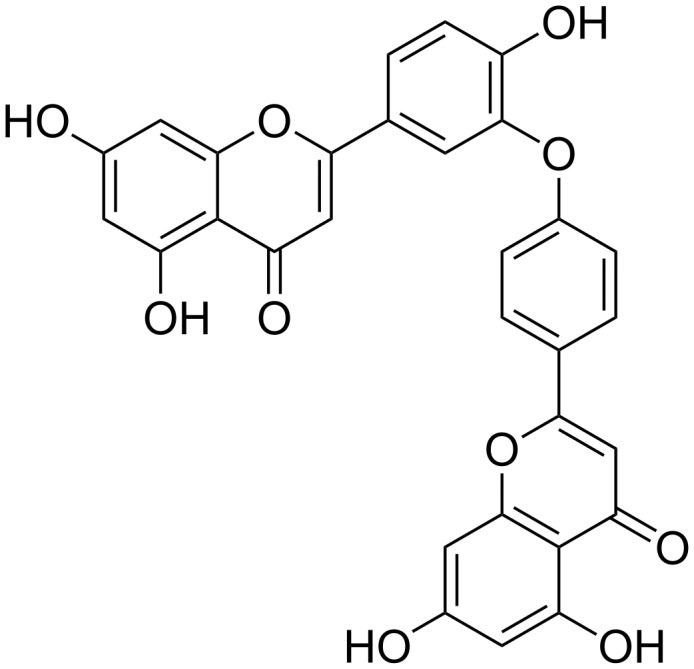
Structure of ochnaflavone (**1**).

Ochnaflavone (**1**) (Figure 1) is a biflavone with an ether linkage between the B-rings of the apigenin and luteolin subunits [12], which has been isolated from several members of the Ochnaceae, a plant family that is rich in biflavonoids [13]. It is considered as the taxonomic marker for the genus *Ochna* [13]. A wide range of activities have been reported for **1**, amongst them prominent anticancer [14–15], anti-inflammatory and atherogenic activity [16–17]. The activity associated with **1** prompted us to develop an efficient synthesis of this compound.

The only previous synthesis of a derivative of **1** was by Okigawa et al. [12,18]. The group synthesized the pentamethyl ether of biflavone **1** by using the Baker–Vankataraman rearrangement as the key step. The conditions used in this reaction sequence were fairly harsh, moderate yields were obtained and the authors did not synthesize the desired product, ochnaflavone (**1**). In this paper we report the synthesis of **1** with aromatic nucleophilic substitution and chalcone formation as the key steps.

## Results and Discussion

**Scheme 1:**
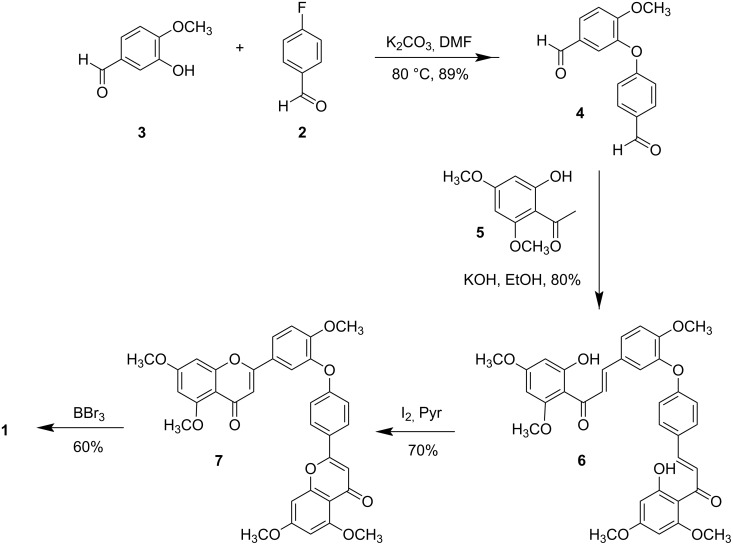
Synthesis of ochnaflavone (**1**).

Our synthesis of **1** is summarized in Scheme 1. The two most important steps in the synthesis of **1** are the formation of the diaryl ether linkage and the assembly of the flavone nuclei. The most logical approach to the synthesis of **1** would be to start with the preparation of a diaryl ether. This functionality is present in many important bioactive natural compounds [19] and it is not surprising that a great amount of effort has been focused on the development and improvement of the methods to form diaryl ethers [20–21]. The Ullman reaction is often used to prepare diaryl ethers, but this reaction has the disadvantages of high reaction temperatures and intolerance to a wide variety of functional groups, with the major weakness being the inconsistency in the products obtained by the use of different copper sources [22–24]. We have opted to prepare the diaryl ether by using an aromatic nucleophilic substitution reaction. 4-Fluorobenzaldehyde (**2**) is a readily available starting material, and under basic conditions it reacted with isovanillin (**3**) to form the diaryl ether **4** in a high yield.

The next step was the formation of the chalcone by an aldol condensation reaction with a suitably substituted acetophenone. Selective methylation of 2,4,6-trihydroxyacetophenone with methyl iodide yielded 2'-hydroxy-4',6'-dimethoxyacetophenone (**5**) in moderate yield. The Claisen–Schmidt condensation of **5** with diaryl ether **4** under basic conditions (KOH) in EtOH proceeded smoothly to furnish dimeric chalcone **6**, which was obtained as a yellow powder.

**Scheme 2:**
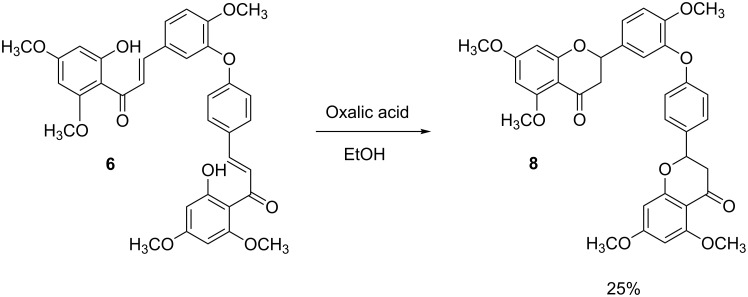
Oxalic acid-induced ring closure of bichalcone **6**.

The cyclization of **6** into the corresponding biflavone **7** was initially attempted by using a catalytic amount of iodine in DMSO at 130–140 °C, conditions often used for the oxidative cyclization of chalcones to flavones [25]. However, at this temperature, the reaction resulted in decomposition of the starting material. The reaction was repeated under microwave conditions as described by Menezes et al. [26] to decrease the time in which **6** was exposed to a high temperature, but decomposition was still observed. The reaction was repeated under same conditions (DMSO/I_2_) at various temperatures, and it was observed that at lower temperatures, a longer time was taken for the starting material to be consumed, but it still resulted in a mixture of products. The cyclization reaction was repeated with DDQ in dry dioxane under reflux (101 °C) [27], but the starting material again decomposed, and when the temperature was lowered to 80 °C, a mixture of products was observed. It was clear that the ether-linked dimeric chalcone decomposes at temperatures exceeding 100 °C. Reaction of dimeric chalcone **6** with oxalic acid as a catalyst in absolute ethanol at 80 °C for 24 h as described by Zambare et al. [28] did not yield the desired compound **7** and instead an ether-linked pentamethoxybiflavanone **8** was obtained. After 72 h, the starting material could still be observed on TLC due to the interconvertibility of the two compounds through chalcone–flavanone equilibrium [29–30] (Scheme 2). Biflavanone **8** is the permethyl ether of 2,3,2'',3''-tetrahydroochnaflavone, a natural product isolated from the New Zealand tree *Quintinia acutifolia*, which is cytotoxic against P388 murine lymphocytic leukemia cells [31]. The synthesis of this compound has not been reported previously.

The desired biflavone **7** was finally obtained in good yield by treating the dimeric chalcone with a catalytic amount of iodine in pyridine. Ochnaflavone (**1**) was obtained as a yellow solid by the reaction of **7** with boron tribromide in dry CH_2_Cl_2_ and had the same spectroscopic properties as the product isolated in our laboratory from the leaves of *Ochna serrulata*.

## Conclusion

In conclusion, we have managed to optimize conditions for the preparation of ochnaflavone (**1**) in a synthetic sequence that is amenable to the synthesis of derivatives of **1**. The synthesis of the pentamethyl ether of a natural-occurring cytotoxic 2,3,2'',3''-tetrahydroochnaflavone was also achieved, albeit in a low yield.

## Experimental

**3-(4-Formylphenyloxy)-4-methoxybenzaldehyde (4)** [32]**:** To a mixture of isovanillin (**3**) (500 mg, 3.29 mmol) and anhydrous potassium carbonate (681 mg, 4.93 mmol) in 15 mL dry DMF under nitrogen, 4-fluorobenzaldehyde (**2**) (448 mg, 3.61 mmol) was added. The mixture was heated at 80 °C under stirring until all the starting material had been consumed (monitored by TLC). The reaction was then left to cool to room temperature, 20 mL cold water was added, and the mixture was extracted with 3 × 20 mL CHCl_3_. The organic layer was dried over anhydrous magnesium sulfate, and the solvent was removed in vacuo. The residue obtained was purified by silica-gel column chromatography (EtOAc/hexanes, 3:7) as eluent to yield 748 mg (89%) of **4** as a light yellow solid.

^1^H NMR (400 MHz, CDCl_3_) δ_H_ 9.94 (s, 1H), 9.93 (s, 1H), 7.89 (d, *J* = 8.8 Hz, 2H), 7.89 (dd, *J* = 8.5, 2.0 Hz, 1H), 7.69 (d, *J* = 2.0 Hz, 1H), 7.38 (d, *J* = 8.5 Hz, 1H), 7.06 (d, *J* = 8.8 Hz, 2H), 3.91 (s, 3H); ^13^C NMR (100 MHz, CDCl_3_) δ_C_ 190.6, 190.0, 162.6 (2C), 156.9, 143.4, 131.9 (2C), 130.9, 129.5, 122.3, 116.3 (2C), 113.4, 55.9; HRMS–ESI (positive-ionization mode): *m*/*z* 279.0625 [M + Na]^+^ (calculated for C_15_H_12_O_4_Na, 279.0633).

**2'-Hydroxy-4',6'-dimethoxyacetophenone (5)** [33]**:** To a well-stirred mixture of 2',4',6'-trihydroxyacetophenone (500 mg, 2.97 mmol) and methyl iodide (1.06 g, 7.47 mmol) in 20 mL acetone, 1.02 g (7.38 mmol) of anhydrous potassium carbonate was added. The reaction mixture was heated under reflux for 5 h while being monitored by TLC until all the starting material had been consumed. The reaction mixture was left to cool and 40 mL of cold acidified distilled water was added, followed by extraction with 3 × 20 mL CHCl_3_. The obtained organic layer was dried over anhydrous magnesium sulfate, and the solvent was removed in vacuo. The residue was purified by silica gel column chromatography (EtOAc/hexanes, 1:9) to afford **5** (291 mg, 50%) as a white solid.

^1^H NMR (400 MHz, CDCl_3_) δ_H_ 13.99 (s, 1H), 6.02 (d, *J* = 2.1 Hz, 1H), 5.89 (d, *J* = 2.1 Hz, 1H), 3.83 (s, 3H), 3.79 (s, 3H), 2.58 (s, 3H); ^13^C NMR (100 MHz, CDCl_3_) δ_C_ 203.1, 167.5, 166.1, 162.9, 105.9, 93.5, 90.6, 55.5 (2C), 32.9; HRMS–ESI (negative-ionization mode): *m*/*z* 195.0662 (calculated for C_10_H_11_O_4_, 195.0657).

**Synthesis of ether-linked dimeric chalcone 6:** To a solution of 404 mg (1.58 mmol) of diaryl ether **4** in EtOH (50 mL), 620 mg (3.16 mmol) of 2'-hydroxy-4',6'-dimethoxyacetophenone (**5**) was added. The solution was cooled in an ice bath followed by the addition of powdered KOH (353 mg, 6.29 mmol). After overnight stirring, the reaction mixture was diluted with ice-cold water (50 mL) and acidified with 6 M HCl. The formed **6** precipitated out as a yellow solid and was filtered off under reduced pressure and washed with water. Product **6** (772 mg, 80%) was obtained as a yellow solid.

^1^H NMR (400 MHz, CDCl_3_) δ_H_ 14.34 (s, 1H), 14.31 (s, 1H), 7.85 (d, *J* = 15.7 Hz, 1H), 7.79 (d, *J* = 15.7 Hz, 1H), 7.73 (s, 2H), 7.59 (d, *J* = 8.5 Hz, 2H), 7.42 (dd, *J* = 8.2, 2.0 Hz, 1H), 7.33 (d, *J* = 2.0 Hz, 1H), 7.05 (d, *J* = 8.2 Hz, 1H), 7.0 (d, *J* = 8.5 Hz, 2H), 6.12 (d, *J =* 2.2 Hz, 1H), 6.10 (*J =* 2.2 Hz, 1H), 5.97 (d, *J =* 2.2 Hz, 1H), 5.94 (d, *J =* 2.2 Hz, 1H), 3.9 (s, 3H), 3.89 (s, 3H), 3.85 (s, 3H), 3.83 (s, 6H); ^13^C NMR (100 MHz, CDCl_3_) δ_C_ 192.5, 192.3, 168.4 (2C), 166.2 (2C), 162.5 (2C), 159.4, 153.1, 144.6, 141.8, 141.4, 130.3, 130.0 (2C), 129.2, 126.9, 126.2 (2C), 120.2, 117.4 (2C), 112.9, 106.4, 106.3, 93.9 (2C), 91.3, 91.2, 56.1, 55.8, 55.6 (2C); HRMS–ESI (positive-ionization mode), *m*/*z* 635.1888 [M + Na]^+^ (calculated for C_35_H_32_O_10_Na, 635.1893).

**Pentamethoxybiflavanone 8:** To a mixture of oxalic acid (10 mol %) and **6** (400 mg, 0.65 mmol), 20 mL of absolute ethanol was added. The stirred reaction mixture was heated at 80 °C for 72 h while the reaction was monitored by TLC. The reaction mixture was then cooled to room temperature, and the solvent was evaporated under reduced pressure. This was followed by extraction with 50 mL of water and 3 × 30 mL CH_2_Cl_2_, and the organic layer thus collected was dried over anhydrous magnesium sulfate. CH_2_Cl_2_ was removed in vacuo and the resulting residue was purified by silica-gel column chromatography with EtOAc/hexanes (1:1) to afford 100 mg (25%) of the ether-linked biflavanone **8** as a light-yellow solid.

^1^H NMR (400 MHz, CDCl_3_) δ_H_ 7.40 (d, *J* = 8.5 Hz, 2H), 7.26 (dd, *J* = 8.5, 2.0 Hz, 1H), 7.16 (d, *J* = 2.0 Hz, 1H), 7.06 (d, *J* = 8.5 Hz, 1H), 6.99 (d, *J* = 8.5 Hz, 2H), 6.16 (d, *J* = 2.2 Hz, 1H), 6.14 (d, *J* = 2.0 Hz, 1H), 6.10 (d, *J* = 2.0 Hz, 2H), 5.37 (m, 2H), 3.9 (s, 6H), 3.87 (s, 3H), 3.83 (s, 6H), 3.02 (m, 2H), 2.8 (m, 2H); HRMS–ESI (positive-ionization mode), *m*/*z* 635.1887 [M + Na]^+^ (calculated for C_35_H_32_O_10_Na, 635.1893).

**5,5'',7,7'',4'-Penta-*O*-methylochnaflavone (7)** [12]**:** To a stirred solution of dimeric chalcone **6** (600 mg, 0.98 mmol) in 15 mL pyridine, 498 mg (1.93 mmol) of iodine was added and the mixture was heated to 80 °C while being stirred for 24 h. The reaction was left to cool to room temperature, and a cold solution of sodium thiosulfate was added to the reaction mixture until all excess iodine had been consumed. This was followed by extraction with 3 × 30 mL CH_2_Cl_2_, the obtained organic layer was washed with 30 mL of water and dried over anhydrous magnesium sulfate. The solvent was removed in vacuo and the crude reaction mixture was purified by silica-gel column chromatography with EtOAc/hexanes (8:2) to obtain **7** (417 mg, 70%) as a yellow solid.

^1^H NMR (400 MHz, CDCl_3_) δ_H_ 7.82 (d, *J* = 8.8 Hz, 2H), 7.73 (dd, *J* = 8.7, 2.3 Hz, 1H), 7.61 (d, *J* = 2.3 Hz, 1H), 7.12 (d, *J* = 8.7 Hz, 1H), 7.02 (d, *J* = 8.8 Hz, 2H), 6.54 (d, *J* = 2.2 Hz, 1H), 6.51 (d, *J* = 2.4 Hz, 1H), 6.36 (d, *J* = 2.2 Hz, 1H), 6.35 (d, *J* = 2.4 Hz, 1H), 6.59 (s, 1H), 6.55 (s, 1H), 3.94 (s, 3H), 3.93 (s, 3H), 3.89 (s, 3H), 3.88 (s, 3H), 3.87 (s, 3H); ^13^C NMR (100 MHz, CDCl_3_) δ_C_ 177.5, 177.4, 164.0, 164.0, 160.9, 160.3, 160.3, 159.8, 159.7, 159.6, 154.0, 143.8, 127.7, 125.8, 124.7, 124.0, 119.8, 116.6, 113.0, 109.2, 109.1, 108.2, 96.2, 96.1, 92.8, 56.4, 56.1, 55.7; HRMS–ESI (positive-ionization mode), *m*/*z* 631.1573 [M + Na]^+^ (calculated for C_35_H_28_O_10_Na, 631.1580).

**Ochnaflavone (1)** [12]**:** A solution of boron tribromide (206 mg, 0.82 mmol) in 5 mL dry CH_2_Cl_2_ was slowly added with a syringe to a stirred solution of 40 mg (0.07 mmol) **7** in 10 mL CH_2_Cl_2_ under nitrogen. After complete addition of BBr_3_, the reaction mixture was stirred at room temperature for 72 h, and then poured into 30 mL ice water. The mixture was then shaken to hydrolyse the excess BBr_3_ and boron complexes; a phenolic product was obtained by extraction with 3 × 20 mL EtOAc. The combined organic layers was dried over anhydrous magnesium sulfate and the solvent was removed in vacuo to obtain a dry crude reaction mixture. Purification of the reaction mixture by silica-gel column chromatography with MeOH/CHCl_3_ (1:9) afforded 21.2 mg (60%) of **1** as a yellow solid.

^1^H NMR (400 MHz, (CD_3_)_2_CO) δ_H_ 12.95 (s, 1H), 12.94 (s, 1H), 8.08 (d, *J* = 8.9 Hz, 2H), 7.90 (dd, *J* = 8.5, 2.1 Hz, 1H), 7.89 (d, *J* = 2.1 Hz, 1H), 7.29 (d, *J* = 8.5 Hz, 1H), 7.16 (d, *J* = 8.9 Hz, 2H), 6.73 (s, 2H), 6.56 (d, *J* = 2.0 Hz, 1H), 6.55 (d, *J* = 2.4 Hz, 1H), 6.28 (d, *J* = 2.0 Hz, 1H), 6.27 (d, *J* = 2.0 Hz, 1H); ^13^C NMR (100 MHz, (CD_3_)_2_CO) δ_C_ 182.2, 182.2, 164.2, 164.18, 163.5, 163.1, 162.5, 161.1, 157.9 (2C), 154.1, 142.4, 128.3 (2C), 125.4, 123.6, 120.7, 118.1, 116.6 (2C), 104.3, 104.52, 104.5, 104.0, 98.9 (2C), 93.9 (2C); HRMS–ESI (negative ionization mode): *m*/*z* 537.0826 (calculated for C_30_H_17_O_10_, 537.0822).
